# Deregulated SLC2A1 Promotes Tumor Cell Proliferation and Metastasis in Gastric Cancer

**DOI:** 10.3390/ijms160716144

**Published:** 2015-07-16

**Authors:** Shiyan Yan, Yuqin Wang, Meimei Chen, Guangming Li, Jiangao Fan

**Affiliations:** Department of Gastroenterology, Xinhua Hospital, Shanghai Jiaotong University School of Medicine, Shanghai 200092, China; E-Mails: shiyan871111@gmail.com (S.Y.); yuqingwang02@gmail.com (Y.W.); chenmeimei27@gmail.com (M.C.); liguangming@gmail.com (G.L.)

**Keywords:** SLC2A1, GLUT1, gastric cancer, proliferation, metastasis

## Abstract

Gastric cancer (GC) is one of the common reasons of cancer-related death with few biomarkers for diagnosis and prognosis. Solute carrier family 2 (facilitated glucose transporter) member 1 protein SLC2A1, also known as glucose transporter type 1 (GLUT1), has been associated with tumor progression, metastasis, and poor prognosis in many human solid tumors. However, little is reported about its clinical significance and biological functions in GC. Here we observed a strong up-regulation of SLC2A1 in patients with GC and found that SLC2A1 was significantly correlated with depth of invasion and clinical stage. Additionally, over-expression of SLC2A1 in GC cells promotes cellular proliferation and metastasis *in vitro* and enhances tumor growth *in vivo* as well as enhancement of glucose utilization. Meanwhile, elevated SLC2A1 also contributes to tumor metastasis *in vitro*. Our results indicate SLC2A1 exhibits a pivotal role in tumor growth, metastasis and glucose metabolism, and also suggest SLC2A1 as a promising target for gastric cancer therapy.

## 1. Introduction

Gastric cancer (GC) is one of the most common malignancies worldwide, with considerable incidence variation across various countries and contributes significantly to cancer mortality [1,2]. Although the traditional therapeutic strategies are used in patients with GC treatment, they are not effective enough to destroy GC cell thoroughly [3]. Recently, it has been reported that several regulators of the glycolysis pathway are detected in various premalignant lesions and tumors, which suggests that these proteins participate in early carcinogenesis and progression of cancer [4,5]. Aerobic glycolysis, also known as Warburg effect, not only favors tumor cell proliferation by providing cellular building blocks, but also contributes to tumor cell metastasis by acidified microenvironment through increased production of lactate [6]. Therefore, glycolysis pathway might be a potential targetable pathway for cancer therapy because of the glucose addiction of tumor cells.

Solute carrier family 2 facilitated glucose transporter member 1 (SLC2A1), also known as glucose transporter 1 (GLUT1), is a crucial protein in the cellular energy metabolism pathway [7,8,9]. SLC2A1 is over-expressed in several different types of carcinomas, including liver, lung, endometrial, oral and breast cancers, as well as gastric cancer [10,11,12,13,14]. These observations suggest that SLC2A1 could be one of the driver genes in tumors. However, the expression pattern and cellular functions of SLC2A1 in GC remain largely unexplored.

In our study, we observed that SLC2A1 was over-expressed in GC tumors by immunochemical staining (IHC) and found the significant correlation between SLC2A1 expression and depths of invasion and clinical stage. We further explored the functional significance of SLC2A1 in GC tumorigenesis and demonstrated that SLC2A1 promoted GC cells growth *in vitro* and *in vivo* and enhanced GC cells migration *in vitro* via enhancement of glucose utilization.

## 2. Results

### 2.1. Solute Carrier Family 2 Facilitated Glucose Transporter Member 1 (SLC2A1) Is Over-Expressed in Gastric Cancer (GC) Tissues

**Figure 1:**
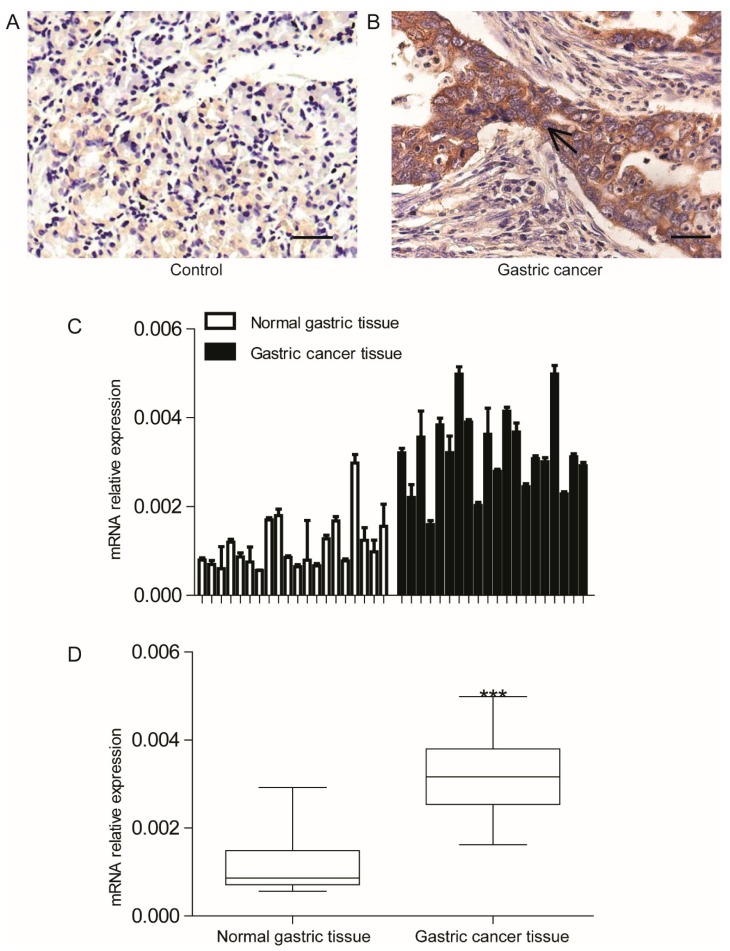
Solute carrier family 2 facilitated glucose transporter member 1 (SLC2A1) was over-expressed in GC tissues. Representative photographs of SLC2A1 immunoreactivity in gastric cancer (GC) tissues (**A**) and normal gastric mucosa (**B**) (scale bar: 50 μm). The arrow represents cytoplasmatic staining of SLC2A1 in tumor cells; (**C**,**D**) SLC2A1 expression in 20 pairs of GC tissues and their normal counterparts was demonstrated by real-time PCR. Normal gastric tissue *vs.* GC tissues, *** *p* < 0.001.

To determine the role of SLC2A1 in GC cells, immunohistochemical staining was performed to confirm the presence of this enzyme. In 57.8% (115/199) of the GC tumors, a predominantly cytoplasmatic staining with strong positive tumor cells for SLC2A1 was detected (Figure 1A), whereas 12.5% (1/8) normal gastric specimens showed only weak irregular cytoplasmatic staining (Figure 1B). To validate the up-regulation of SLC2A1 at the transcriptional level in GC, we further compared SLC2A1 expression patterns in 20 GC tumor tissues to their normal counterparts using real-time PCR. Our data revealed that the mean expression level for SLC2A1 in GC tumor tissues was roughly 3-fold higher than that in the normal gastric mucosa (*p* < 0.001, Figure 1C,D). Furthermore, we analyzed the correlation of SLC2A1 expression with the clinicopathological features. SLC2A1 expression was significantly correlated with depths of invasion (*p* = 0.011) and clinical stage (*p* = 0.001) (Table 1). Collectively, these findings suggest that SLC2A1 is over-expressed in GC tissues and might contribute to the progression of GC.

**Table 1:** Analysis of SLC2A1 expression with corresponding clinical parameters.

Characteristics		Cases	SLC2A1 Expression		*p* Value
			Low	High	
Normal gastric tissue		8	7	1	0.023
Gastric cancer tissue		199	84	115	
T classification	T1	14	10	4	0.011
	T2	130	59	71	
	T3	48	14	34	
	T4	7	1	6	
Clinical stage (AJCC)	I	134	69	65	0.001
	II	50	11	39	
	III	12	3	9	
	IV	3	1	2	

### 2.2. SLC2A1 Promotes GC Cells Growth in Vitro and in Vivo

**Figure 2:**
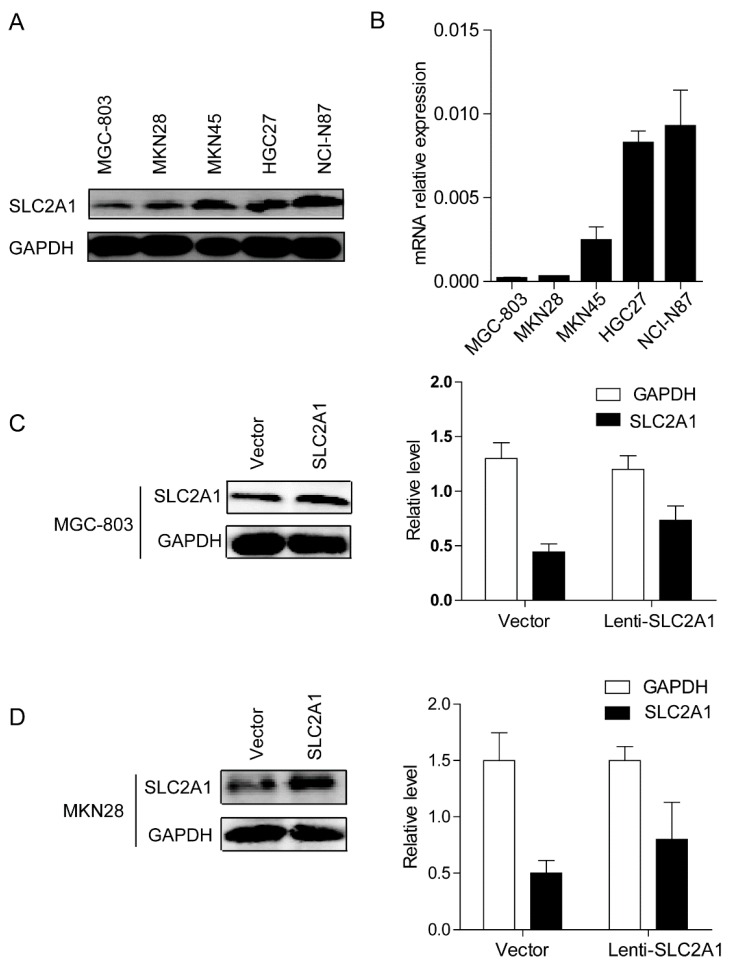
SLC2A1 expression in GC cell lines. The protein (**A**) and mRNA (**B**) levels of SLC2A1 in different gastric cancer cell lines; (**C**) The mRNA and protein levels of SLC2A1 in SLC2A1-expressing MGC-803 cells; (**D**) The mRNA and protein levels of SLC2A1 in SLC2A1-expressing MKN28 cells.

**Figure 3:**
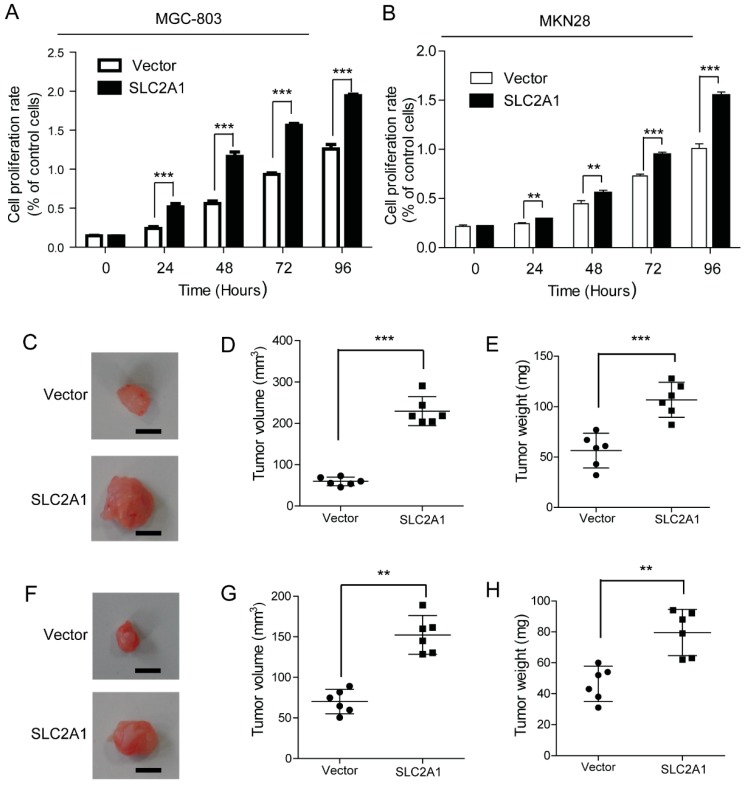
SLC2A1 promoted GC cell growth *in vitro* and *in vivo*. Cell counting kit-8 (CCK-8) assay showed over-expression of SLC2A1 promoted cell growth in MGC-803 (**A**) and MKN28 cells (**B**); (**C**–**E**) The mean tumor volume and weight in the mice inoculated with SLC2A1- and vector-expressing MGC-803 cells (*n* = 6); (**F**–**H**) The mean tumor volume and weight in the mice inoculated with SLC2A1- and vector-expressing MKN28 cells (*n* = 6). Vector *vs.* SLC2A1, ** *p* < 0.01, *** *p* < 0.001; Scale bar in (**C**,**F**): 5 mm.

We first examined the mRNA (Figure 2A) and protein (Figure 2B) level of ectopic SLC2A1 expression in several GC cells and found that MGC-803 and MKN28 cells expressed relatively lower endogenous levels of SLC2A1. To further characterize the functions of SLC2A1, we established stable cell lines over-expressing SLC2A1 in MGC-803 and MKN28 cells, and the transfection efficiency of SLC2A1 in the stable cell lines was verified by western blot (Figure 2C,D). Cell counting kit-8 (CCK-8) assay showed significant increases in MGC-803cellular proliferation after transfecting the SLC2A1 plasmid (Figure 3A). Similarly, the MKN28 cells transfected with the SLC2A1 plasmid grew faster than that transfected with empty vector (Figure 3B). Next, we subcutaneously injected the SLC2A1- or vector-expressing MGC-803 into nude mice (Figure 3C). After four weeks, mice were sacrificed. The mice injected with the SLC2A1-expressing MGC-803 cells demonstrated a significantly larger in tumor size than those injected with the vector-expressing MGC-803 cells (*p* < 0.001, Figure 3D). The mice injected with the SLC2A1-expressing MGC-803 cells also demonstrated a significant increase in tumor weight than those injected with the vector-expressing MGC-803 cells (*p* < 0.001, Figure 3E). Similarly, the mice injected with the SLC2A1-expressing MKN28 cells demonstrated a significant larger in tumor size and tumor weight than those injected with the vector-expressing MKN28 cells (*p* < 0.01, Figure 3F–H). Taken together, these results support the role of SLC2A1 in promoting growth of GC cells *in vitro* and *in vivo*.

### 2.3. SLC2A1 Enhances GC Cells Invasion in Vitro

**Figure 4:**
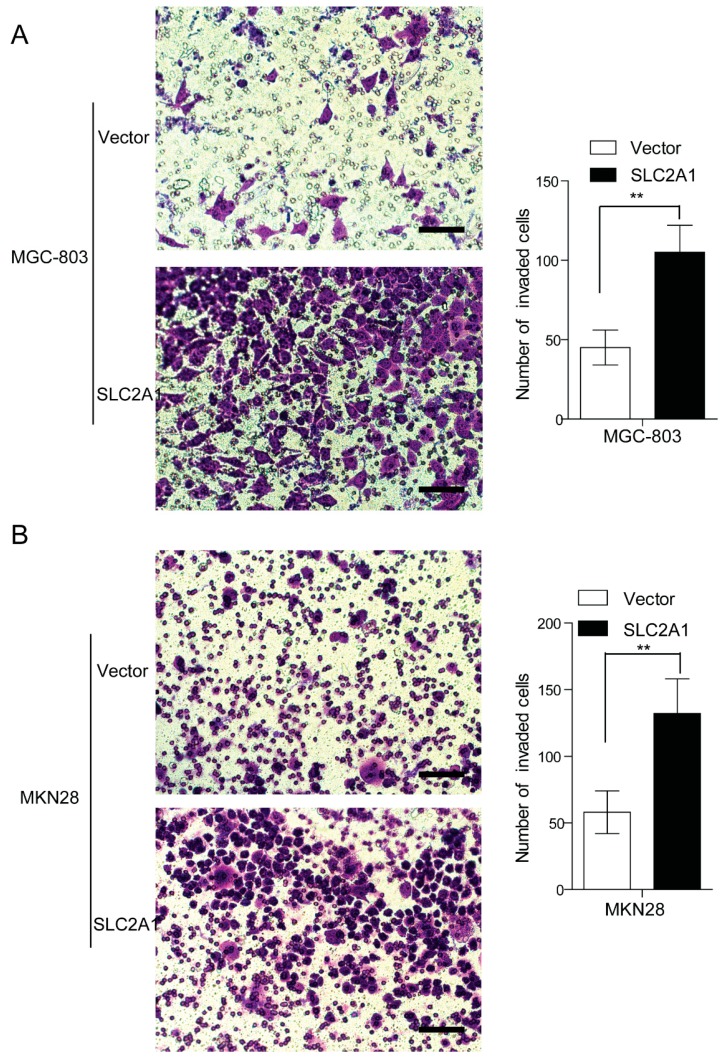
SLC2A1 enhanced GC cells invasion *in vitro*. The invasive potential of SLC2A1- and vector-expressing MGC-803 (**A**) and MKN28 cells (**B**) was assessed by Transwell model. Vector *vs.* SLC2A1, ** *p* < 0.01; Scale bar: 50 μm.

As elevated SLC2A1 is closely associated with depth of invasion and clinical stage, we hypothesized that over-expression of SLC2A1 may facilitate tumor cell invasion and further contribute to distant metastasis. Thus, we investigated the effect SLC2A1 on GC cells invasion. By Transwell model, we observed that more numbers of invaded cells in SLC2A1-expressing group compared with the control group in both MGC-803 (*p* < 0.01, Figure 4A) and MKN28 (*p* < 0.01, Figure 4B) cells. These data suggest that over-expression of SLC2A1 provides an advantage for gastric cancer metastasis *in vitro*.

### 2.4. SLC2A1 Promotes GC Cells Glucose Utilization in Vitro

**Figure 5:**
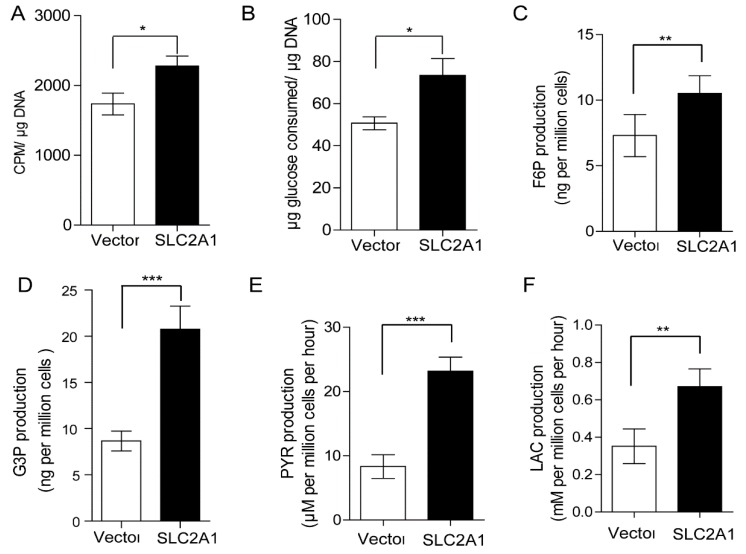
SLC2A1 promoted glucose utilization of GC cells. (**A**) SLC2A1-expressing MGC-803 cells took more 3H-2-deoxyglucose (3H-2-DOG) compared with the control cells; (**B**) MGC-803 cells expressing SLC2A1 consumed more glucose than controls; (**C**) The quantitative changes of fructose-6-phosphate (F6P) in SLC2A1-expressing MGC-803 cells compared with that in vector expressing cells; (**D**) The quantitative changes of G3P in SLC2A1-expressing MGC-803 cells compared with that in vector expressing cells; (**E**) The production level of pyruvate in the culture buffer of SLC2A1- and vector-expressing MGC-803 cells; (**F**) The production level of lactate in the culture buffer in SLC2A1- and vector-expressing MGC-803 cells. Vector *vs.* SLC2A1, * *p* < 0.05, ** *p* < 0.01, *** *p* < 0.001.

Next, we sought to determine whether the GC cells with increased SLC2A1 protein had altered glucose utilization. As shown in Figure 5A, SLC2A1-expressing MGC-803 cells took more 3H-2-deoxyglucose (3H-2-DOG) compared to the control cells (*p* < 0.05). Similarly, MGC-803 cells expressing SLC2A1 consumed more glucose than the negative control (*p* < 0.05, Figure 5B). Meanwhile, we found an increase in fructose-6-phosphate (*p* < 0.01, Figure 5C) and glyceraldehydes-3-phosphate production (G3P) (*p* < 0.001, Figure 5D) in SLC2A1-expressing MGC-803 cells compared with vehicle-expressing cells. Furthermore, we found significantly increased pyruvate production in SLC2A1-expressing MGC-803 cells compared with vehicle-expressing cells (*p* < 0.001, Figure 5E). Similarly, there’s an increase in lactate production in SLC2A1-expressing MGC-803 cells compared with vehicle-expressing cells (*p* < 0.01, Figure 5F). These data demonstrate that increase in SLC2A1 expression leads to an enhancement of glucose transport, glucose consumption and lactate secretion, indicating the critical roles of SLC2A1 in glycolysis and glucose metabolism. Overall, these data supported that GC cells with increased SLC2A1 protein had enhanced glucose utilization.

## 3. Discussion

Gastric cancer (GC) patients are diagnosed at a late stage with a poor prognosis, because of lack of reliable markers for early detection [15,16,17] and the mechanisms of GC is still unclear. *SLC2A1* gene encodes GLUT1 protein and is one of the isoforms of the protein family of facilitative glucose transporters, which is highly concentrated in tissue endothelium and epithelium [18,19]. Recently, accumulated evidences demonstrated that high expression of SLC2A1 involves in the development of various carcinomas [20,21,22].

Previous immunohistochemical studies showed that SLC2A1 was highly expressed in about 25% of detected samples [23,24]. In breast cancer, SLC2A1 expression was closely correlated with higher tumor grade [25]. In our study, we observed that SLC2A1 was over-expressed in GC tissues, in relative to that in normal gastric tissues, and the high rate of GC tissues is 57.8%. It was also demonstrated that SLC2A1 over-expression was associated with several clinicopathologic parameters of endometrium and endometrial adenocarcinoma [26]. Consistent with this, our results indicated that SLC2A1 expression significantly correlated with depth of invasion and clinical stage. However, two researches in GC focused on SLC2A1 study demonstrated that none of normal gastric epithelium expressed SLC2A1 and nearly 30% carcinoma samples were positive staining of SLC2A1 [27,28]. These minor data differences might due to different scoring criteria in each study.

Up-regulated SLC2A1 expression contributes to carcinogenesis and tumor progression [29]. Next, we analyzed the oncogenic activity of SLC2A1 in GC cell lines. We established stable cell lines over-expressing SLC2A1 in MGC-803 and MKN28 cells to further explore the function of SLC2A1. Firstly, we observed that MGC-803 and MKN28 cells with exogenous SLC2A1 over-expression grew faster than control cells. Besides, we showed that SLC2A1-over-expressing MGC-803 and MKN28 cells grew faster and larger tumor xenografts than control cells. Secondly, we found over-expression of SLC2A1 provided an advantage for GC cells to invasion. Finally, SLC2A1-expressing MGC-803 cells took more 3H-2-DOG, consumed more glucose and produced more fructose-6-phosphate, glyceraldehydes-3-phosphate, pyruvate and lactate compared with vector-expressing MGC-803 cells. And consistent with this observation, it was reported that glyceraldehydes-3-phosphate was increased in disease progression from benign tissue to prostate cancer [30]. Given the roles of SLC2A1 in enhancing glucose utilization, thus supporting energy requirements, providing enormous biosynthetic needs and promoting the secretion of lactate and further contributing to the acidification of tumor microenvironment, which ultimately favors tumor growth and progression, it is reasonable to expect the oncogenic functions in gastric cancer.

In summary, our study provides evidence that elevated SLC2A1 expression is significantly correlated with depth of invasion and clinical stage. Meanwhile, over-expressed SLC2A1 promotes the proliferation, invasion and glucose utilization of GC cells. These results suggest that SLC2A1 might acts as a candidate target for developing treatment of GC.

## 4. Experimental Section

### 4.1. Cell Culture

The human gastric cancer cell lines (MGC-803, MKN28, MKN-45, HGC-27 and NCI-N87) were purchased from American Type Culture Collection (ATCC) and maintained in Dulbecco’s modified Eagle’s medium or RPMI 1640 medium (Gibco, Grand Island, NY, USA) supplemented with 10% fetal bovine serum (FBS) and penicillin-streptomycin (50 U/mL) at 37 °C in a atmosphere containing 5% CO_2_.

### 4.2. Immunohistochemistry

Tissue microarray (TMA) contained 199 cases of GC tissues and eight cases of normal gastric tissues were purchased from Xi’an AlenabioInc (Xi’an, China). The clinical stages were classified according the definition by the American Joint Committee on Cancer (AJCC). After deparaffinized with dimethylbenzene and rehydrated through grade ethanol, the gastric carcinomas tissue arrays were treated with 10% BSA in phosphate-buffered saline (PBS) for 15 min and incubated with primary antibody (anti-SLC2A1, dilution at 1:50; Abcam, Shanghai, China) at 37 °C for 45 min. The sections were then washed with Tween and incubated with a universal biotinylated secondary antibody at 37 °C for 30 min. Scoring was conducted by two pathologists, according to the ratio and intensity of positive-staining cells.

### 4.3. Quantitative Reverse Transcriptase-PCR

The collection of human specimens for quantitative reverse transcriptase-PCR study is approved by the ethics committee of Xinhua Hospital, Shanghai Jiaotong University School of Medicine. Total RNA from frozen tissues or cell lines was purified using the TRIzol reagent (Invitrogen, Carlsbad, CA, USA) according to manufacturer’s instructions. Complementary DNA synthesis (RT) was carried out starting with total RNA with Prime Script RT reagent kit (Takara, Dalian, China). The primers used for real-time PCR are as follows. GAPDH forward: 5′-TGACTTCAACAGCGACACCCA-3′, GAPDH reverse: 5′-CACCCTGTTGCTGTAGCCAAA-3′; SLC2A1 forward: 5′-AAGGTGATCGAGGAGTTCTACA-3′, SLC2A1 reverse: 5′-ATGCCCCCAACAGAAAAGATG-3′. The amplifications were performed in 10 μL reaction volumes with a denaturation at 94 °C for 2 min, 40 thermal cycles of 95 °C for 10 s, 60 °C for 34 s and 72 °C for 30 s. Relative expression was determined by normalizing expression of each *C*t value to glyceraldehyde-3-phosphate dehydrogenase (GAPDH) *C*t value and data were analyzed according to the 2^−ΔΔ*C*t^ formula.

### 4.4. Western Blot Analysis

Cells were isolate by adding lysis buffer and an equal amount of protein lysates was separated by 8%–12% SDS-PAGE gel. After that, protein was transferred onto a nitrocellulose membrane. Membranes were blocked for 30 min and washed with Tris buffered saline (TBS) for three times and probed overnight at 4 °C with the antibody against SLC2A1 (Abcam, Shanghai, China) and β-actin (Sigma, Shanghai, China). The membranes were incubated for 1 h at room temperature with a 1:1000 dilution of secondary antibody (Invitrogen, Carlsbad, CA, USA). After that, the membranes were washed with TBS for three times. Finally, the signals were detected by Odyssey Infrared Imaging system (LI-COR Biosciences, Lincoln, NE, USA).

### 4.5. Lentirival Transduction

Lentivirus-containing supernatant was collected at 48 h after transfection in HEK-293T cells with the pLKO.1-TRC (FulenGen, Guangzhou, China) vector and CD510B-GLUT1 and packaging plasmids (Ppack) including VSV, REV and GAG, 0.2 μm filtered, and snap frozen at −80 °C. MGC-803 and MKN28 cells were infected with 2 mL of lentivirus for 8 h. Cells were selected in puromycin (Sigma) for one week.

### 4.6. Cell Proliferation Assay

Cell growth rate was measured by cell counting kit-8 (CCK-8, Dojindo, Shanghai, China). Briefly, 3000 cells per well were seeded into a 96-well plate and cultured overnight. At 24, 48, 72, and 96 h, cell viability was obtained at 450 nm wavelength and each sample was measured in triplicate to ensure quantitative accuracy.

### 4.7. Tumor Xenograft

For *in vivo* tumorigenicity assays, nude mice (6-week-old) were injected subcutaneously with 5 × 10^6^ SLC2A1-expressing or vector-expressing MGC-803 or MKN28 cells in 0.1 mL volume of PBS. Each week after injection, Tumor size was monitored using micrometer calipers, and tumor volume was calculated as follows: Volume = length × (width)^2^/2. After 4 weeks, the mice were sacrificed and the tumors were dissected. Animal experiments were performed in full accordance with the Medicine Institutional Guidelines of Shanghai Jiaotong University.

### 4.8. Cell Invasion Assay

The invasive ability of gastric cancer cells was measured by Transwell model (Coster, Cambridge, MA, USA) according to the manufacturer’s instructions. Briefly, 2 × 10^4^ cells in 100 μL Roswell Park Memorial Institute-1640 (PRMI-1640) medium were seeded into the upper compartment coated with 100 μL matrigel (BD Bioscience, San Diego, CA, USA). The lower chambers were filled with 700 μL of RPMI-1640 medium containing 5% FBS. After the cells were incubated for 48 h, the non-invading cells that remained on the upper surface were removed and fixed with methanol and stained with hematoxylin. Each plate was measured five predetermined fields to ensure quantitative accuracy.

4.9. 3H-2-Deoxyglucose Transport Assay

Cells were incubated in glucose-free Dulbecco’s modified eagle’s medium (DMEM) and then pulsed with 2 µCi of 3H-2-deoxyglucose (~60 pmol) (PerkinElmer, Nanjing, China) for 10 min. The monolayers were quantitated by liquid scintillation counting. The data is presented as counts per minute (CPM) per µg DNA.

### 4.10. Measurement of Pyruvate, Lactate and Glucose

Cells were cultured in 24 well plates for 24 h. Pyruvate and lactate production was measured by the enzymetic method using a commercially available fluorescence-based assay kit (Invitrogen). The glucose in the conditioned media was quantified using the Glucose Assay kit (Sigma). Quantities of glucose consumed were normalized to the DNA content of each well and triplicate samples were analyzed.

### 4.11. Statistical Analysis

The SPSS software program (version 17.0; IBM Corporation, Armonk, NY, USA) was used for statistical analysis and Graphical representations were performed with GraphPad Prism 5 (GraphPad Software Inc., San Diego, CA, USA) software. The chi-square test and student’s *t*-test were used for comparison between groups. All data are presented as the means ± SD and *p* values less than 0.05 were considered statistically significant.
